# Erythromycin treatment hinders the induction of oral tolerance to fed ovalbumin

**DOI:** 10.3389/fimmu.2012.00203

**Published:** 2012-07-18

**Authors:** Sydney E. Lambert, Jeremy M. Kinder, Jenny E. Then, Kelly N. Parliament, Heather A. Bruns

**Affiliations:** Department of Biology, Ball State University,Muncie, IN, USA

**Keywords:** antibiotics, dendritic cells, *Lactobacillus*, probiotics, T regulatory cells, tolerance

## Abstract

The mucosal immune system is constantly exposed to antigen, whether it be food antigen, commensal bacteria, or harmful antigen. It is essential that the mucosal immune system can distinguish between harmful and non-harmful antigens, and initiate an active immune response to clear the harmful antigens, while initiating a suppressive immune response (tolerance) to non-harmful antigens. Oral tolerance is an immunologic hyporesponsiveness to an orally administered antigen and is important in preventing unnecessary gastrointestinal tract inflammation, which can result in a number of autoimmune and hypersensitivity diseases. Probiotics (beneficial intestinal bacteria), T regulatory cells, and dendritic cells (DCs) are all essential for generating tolerance. Antibiotics are commonly prescribed to fight infections and often necessary for maintaining health, but they can disrupt the normal intestinal probiotic populations. There is increasing epidemiologic evidence that suggests that antibiotic usage correlates with the development of atopic or irritable bowel disorders, which often result due to a breakdown in immune tolerance. This study investigated the effect of the antibiotic erythromycin on oral tolerance induction to ovalbumin. The results demonstrated that antibiotic treatment prior to exposure to fed antigen prevents tolerance to that antigen, which may be associated with a reduction in intestinal *Lactobacillus* populations. Furthermore, antibiotic treatment resulted in a significant decrease in the tolerogenic CD11c^+^/CD11b^+^/CD8α^-^ mesenteric lymph node DCs independent of tolerizing treatment. These results provide evidence that antibiotic treatment, potentially through its effects on tolerogenic DCs and intestinal microflora, may contribute to autoimmune and atopic disorders via a breakdown in tolerance and support prior epidemiologic studies correlating increased antibiotic usage with the development of these disorders.

## INTRODUCTION

The mucosal immune system is the first line of defense for the body to eliminate antigens. This system favors the induction of a suppressive immune response, one that is tolerant to antigen instead of mounting an active immune response against it, and promotes the induction of oral tolerance (Brandtzaeg, 2009). The phenomenon of antigenic tolerance in the intestine following antigen feeding was first described by Chase (1946). This immunologic hyporesponsiveness to antigen that is first encountered by oral administration was later described as oral tolerance (Faria and Weiner, 2005; Hanaway, 2006; Strobel and Mowat, 2006). This results in a state of systemic unresponsiveness (or tolerance) to subsequent challenge with the same antigen given in an immunogenic form. It has been demonstrated that oral tolerance cannot be induced in mice lacking mesenteric lymph nodes (MLNs; Kunkel et al., 2003; Chung et al., 2005). Further studies have shown that tolerance is induced exclusively in the MLNs following the migration of antigen-loaded tolerogenic dendritic cells (DCs) to this site (Worbs et al., 2006). DCs have a crucial role in generating tolerance due to their influence on T cell differentiation. Mucosal DCs are the only DCs that preferentially induce the differentiation of T cells into T regulatory cells (Tregs), and are required for oral tolerance induction (Alpan et al., 2001; Roncarolo et al., 2001; Tezuka and Ohteki, 2010).

Tregs are CD4^+^/CD25^+^ T cells that express Foxp3 and suppress immune responses toward self-antigens, allergens, food antigens, and other non-harmful antigens (Sakaguchi et al., 1995; Fontenot et al., 2003; Hori et al., 2003; Chahine and Bahna, 2010). Depleting the CD4^+^/CD25^+^ Treg population results in a variety of immunological autoimmune diseases and also excessive humoral and cellular immune responses to non-self-antigens (Sakaguchi et al., 1995; Ohkura and Sakaguchi, 2010). Antigen encountered in mucosal tissues is acquired by mucosal DCs which then traffic to the MLNs where they present the antigen to T cells, stimulating Treg differentiation and subsequently inducing tolerance to that antigen. Several different subsets of tolerogenic DCs have been identified in the MLNs, all of which express the surface protein CD11c but vary in their expression of other surface proteins (Milling et al., 2010). DCs expressing CD11c^+^/CD11b^+^/CD8α^-^ preferentially secrete the anti-inflammatory cytokine IL-10 and induce Th2 cells (Niess and Reinecker, 2006; Tezuka and Ohteki, 2010; Scott et al., 2011). CD11c^+^ DCs that express CD103^+^ have been shown to travel to the MLNs and produce retinoic acid to induce FoxP3^+^ Tregs. The combination of mucosal lymphoid structures, specialized mechanisms, and unique cellular components are critical for the induction of tolerance.

Probiotics are viable bacteria that are non-pathogenic, resistant to gastric acid and bile destruction, attach to intestinal epithelial tissue, and can colonize the gastrointestinal (GI) tract (Hanaway, 2006). These indigenous intestinal bacteria begin to colonize the mucous membranes and skin epithelia shortly after birth and provide many benefits to the host; they synthesize and excrete vitamins, compete with pathogenic bacteria for intestinal attachment sites and nutrients, and produce toxins that kill foreign organisms (Levy, 2000; Macpherson and Harris, 2004). Probiotics also metabolize complex carbohydrates, aide in digestion, and improve intestinal function by repairing tight junctions, enhancing mucin production, and suppressing intestinal inflammation (Strobel and Mowat, 2006). The two model probiotic genera are *Lactobacillus* and *Bifidobacterium* (Ivanov and Littman, 2011). Studies have demonstrated a role for *Lactobacillus* and *Bifidobacterium* species in suppressing inflammation and enhancing Foxp3^+^ Treg differentiation in both human and murine models (Di Giacinto et al., 2005; Pronio et al., 2008; Livingston et al., 2010). *L. paracasei*, specifically, has been shown to inhibit the production of Th1 and Th2 cytokines and induce a population of CD4^+^ T cells that secrete high levels of transforming growth factor β (TGF-β) and IL-10 (von der Weid et al., 2001). Importantly, probiotics may mediate these effects through their interactions with DCs, as demonstrated by the potent induction of IL-10 secretion by intestinal and blood DCs by *Lactobacillus* and *Bifidobacterium* species in the probiotic formulation VSL#3 (Hart et al., 2004) and the ability of *L. reuteri* and *L. casei* to prime DCs that induce Treg differentiation (Smits et al., 2005). Furthermore, protection again 2,4,6-trinitrobenzenesulfonic acid (TNBS)-induced colitis was conferred by probiotic-treated DCs via a mechanism that required IL-10 and Treg cells (Foligne et al., 2007).

Disruption of the intestinal microflora, particularly depletion of probiotic species, can lead to a variety of consequences such as diarrhea or yeast infections that are common side-effects of antibiotic treatment (Noverr and Huffnagle, 2005). The effect of antibiotic treatment on intestinal microflora extends far past cessation of treatment. Short-term administration of antibiotics that are present in high levels when they reach the intestine can cause alterations in the probiotic populations for up to 2 years. Even when pre-treatment bacterial numbers are reached, the composition of the bacteria is permanently altered (Littman and Pamer, 2011). Importantly, there are epidemiologic studies that suggest antibiotic usage may be linked to more serious health conditions such as allergy and asthma (Droste et al., 2000; Russell et al., 2012) and irritable bowel disorders such as Crohn’s disease (Card et al., 2004). Furthermore, industrialized nations, which have substantial antibiotic use, have a high incidence of allergic disease, while developing countries with low antibiotic usage have a low incidence of allergic disease (Burney et al., 1994; Asher et al., 1995; Beasley et al., 2000).

While there are increasing numbers of clinical and epidemiologic studies on the benefits of probiotics and their influence on mucosal integrity and immune processes, there is little research providing information about the effect of antibiotic treatment on probiotic populations and the subsequent consequence on specific immune responses. It is possible that the development of atopic disorders and irritable bowel disorders following antibiotic treatment results from a breakdown in tolerance, which may occur due to the depletion of probiotic species. The goal of this study was to examine the effect of antibiotic treatment on oral tolerance induction to ovalbumin (OVA). Our results demonstrate that a 7-day course of the broad-spectrum antibiotic erythromycin, immediately followed with a single tolerizing dose of OVA, is sufficient to impair tolerance induction to OVA. Concomitantly, Treg and tolerogenic DC populations in the MLNs were examined to identify alterations that may correlate with the hindrance in tolerance.

## MATERIALS AND METHODS

### MICE

Balb/c mice between the ages of 8 and 12 weeks, bred from mating pairs purchased from The Jackson Laboratory (Bar Harbor, ME, USA), were used for each study. Mice were housed individually in cages and separated into four treatment groups, with no fewer than three mice per group and with equal numbers of each sex between groups. Methods involving mice were approved by the Ball State University Animal Care and Use Committee.

### EXPERIMENTAL OVERVIEW

For each experiment, there were four treatment groups (*n* = 3–4/group for each experiment): NT, OT, NTAb, and OTAb (NT, non-tolerized, non-antibiotic-treated; OT, orally tolerized to OVA, non-antibiotic-treated; NTAb, non-tolerized, antibiotic-treated; OTAb, orally tolerized to OVA, antibiotic-treated). On each of days 1–5, all mice were administered a probiotic solution to establish an intestinal probiotic flora. Mice were allowed to rest for 2 days, and then on day 8 fecal matter was collected from all mice, plated, and feces cultured to determine intestinal microbial numbers pre-antibiotic treatment. On days 8–13, the antibiotic erythromycin was consecutively administered to NTAb and OTAb mice with water given to NT and OT mice. On day 14, a high-dose of OVA was added to the water (OT) or antibiotic solution (OTAb) of the OT and OTAb mice to induce tolerance. On day 15, all treatments were discontinued and fecal matter was collected from all mice, plated, and feces cultured to determine intestinal microbial numbers post-antibiotic treatment. On day 19, five days after OVA treatment, all mice were immunized with OVA, and they were again immunized on day 26. On day 33, all mice were sacrificed and their serum and MLNs isolated.

### FECAL MATTER PLATING AND CULTURING

Fecal matter was collected and plated before and after antibiotic treatments. 0.07 g of feces was collected from each mouse and placed into 700 μL of 0.9% sterile saline solution and homogenized. The particulate matter was removed, and 50 μL of the remaining liquid was plated on de Man–Rogosa–Sharpe (MRS, Sigma, St. Louis, MO, USA) plates, which select for Lactobacilli. Plates were incubated at 37^°^C and 10% CO_2_ for 24 h to facilitate growth of the facultative anaerobes, Lactobacilli. After 24 h, colonies on each plate were counted to assess the effect of the antibiotic treatment on the Lactobacilli probiotic populations.

### PROBIOTIC, ANTIBIOTIC, AND OVALBUMIN TREATMENTS

To ensure an established probiotic population in the GI tract of all mice prior to the start of the experiment, the probiotic capsules Acidophilus with Pectin (The Vitamin Shoppe) were dissolved in 3 mL of 10% sugar water per capsule. About 300 μL of the probiotic solution was administered via syringe feeding (placing tip of a syringe with no needle into the mouth of the mouse and administering the solution drop-wise into the mouse’s mouth) to each mouse for five consecutive days. For all other treatments, mice were fed via intragastric gavage using ball-tipped feeding needles (SouthPointe Surgical Supply, Coral Springs, FL, USA). Mice received 5 mg erythromycin (Sigma) in 200 μL water total volume (antibiotic-treated groups) or water alone (control) daily for seven consecutive days. On day 7, 20 mg of OVA (Sigma) was added to the 200 μL antibiotic or water solution and administered only to the treatment groups that were to be tolerized to OVA.

### IMMUNIZATIONS

Five days after ceasing control and experimental feeding treatments, all mice were immunized intraperitoneally with 0.1 mg of OVA (Sigma) prepared in phosphate buffered saline (PBS) with equal parts alum (Thermo Scientific) to stimulate an immune response. The final injection volume was 200 μL. One week after the initial immunization, all mice were again immunized with 0.1 mg of OVA.

### ENZYME-LINKED IMMUNOSORBENT ASSAY

One week following the second immunization, all mice were sacrificed by CO_2_ asphyxiation. Blood was collected by cardiac puncture and blood serum was isolated. Levels of OVA-specific IgG present in the serum were detected using an indirect enzyme-linked immunosorbent assay (ELISA). Plates were coated with 400 μg/100 μL/well OVA (Sigma) in coating buffer (Bethyl Laboratories, Montgomery, TX, USA) for 2 h at room temperature (RT). Plates were washed three times in wash buffer (Bethyl Laboratories) between every step. Plates were then blocked for 30 min with blocking solution (Bethyl Laboratories). Serum from each mouse was diluted 1:100, 1:5000, 1:2500, and 1:12,500 for the IgG ELISA, and 1:10, 1:100, and 1:1000 for the IgE ELISA. Samples were then added to the plate, and incubated for 2 h at RT. Rat anti-mouse IgG-AP or rat anti-mouse IgE-AP, human adsorbed (Southern Biotech, Birmingham, AL, USA) was diluted 1:1000 and used for detection of the appropriate antibodies. Samples were analyzed in duplicate using a microplate reader (Model 680, Bio-Rad).

### TISSUE HARVESTING AND CELLULAR ISOLATION

Following cardiac puncture, MLNs were harvested from each mouse and homogenized in RPMI-1640 (Sigma) supplemented with 10% heat-inactivated FBS (Atlanta Biologicals), penicillin–streptomycin, sodium pyruvate, non-essential amino acids, L-glutamine, HEPES, and 5 × 10^-5^ M 2-mercaptoethanol (all from Sigma Chemical). The resulting cell suspensions were washed in RPMI-1640 complete medium, and then red blood cell lysis was performed for 5 min, followed by two washes in RPMI-1640. Cells were then immediately re-suspended in FACS buffer (1× PBS with 2% BSA and 0.1% NaN_3_) and immediately stained for flow cytometric analysis.

### FLOW CYTOMETRY

Multi-color flow cytometric analysis was performed to identify populations of DCs and Tregs in the MLNs. To enumerate DC populations, cells isolated from MLNs were resuspended in FACS buffer. Cells were then blocked with 15 μg rat serum in 5 μL FACS buffer for 5 min at 4^°^C. All staining was done for 10–15 min at 4^°^C followed by two washes with FACS buffer, and then fixed in PBS with 2% BSA, 0.1% NaN_3_ and 0.5% formaldehyde (fix buffer). Samples were incubated with antibodies (specific for the cell surface proteins described in each experiment) conjugated to FITC, PE, or CyChrome (eBioscience, BioLegend, or BD Bioscience as antibody specificity and desired fluorochrome conjugates were available). To enumerate Tregs, cells isolated from MLNs were stained using a Treg identification kit (eBioscience) according to the manufacturer’s instructions. Cells were analyzed on an Accuri C6 flow cytometer using CFlow Plus software (Accuri, Ann Arbor, MI, USA).

### STATISTICAL ANALYSES

All statistical analyses were performed using Sigma Plot (Systat Software, San Jose, CA, USA). Results are represented as mean ± SEM. To determine significant decreases in intestinal bacteria following antibiotic treatment, a Student’s paired *t*-test was performed on pre- and post-treatment colony counts. To determine statistically significant differences in levels of OVA-specific antibodies, data from the ELISAs were analyzed by one-way ANOVA followed by Student–Newman–Keuls *post hoc* analysis. To determine statistically significant differences in percentages of DCs and Tregs present in MLNs, the data from flow cytometric analyses were analyzed by one-way ANOVA followed by Student–Newman–Keuls *post hoc* analysis or Kruskal–Wallis ANOVA on ranks. Differences between groups were considered statistically significant at *p* ≤ 0.05.

## RESULTS

The goal of this study was to demonstrate the effect of antibiotic usage on oral tolerance induction and tolerogenic immune populations in the MLNs using OVA as the antigenic stimulus.

### ANTIBIOTIC TREATMENT HINDERS ORAL TOLERANCE INDUCTION

**FIGURE 1:**
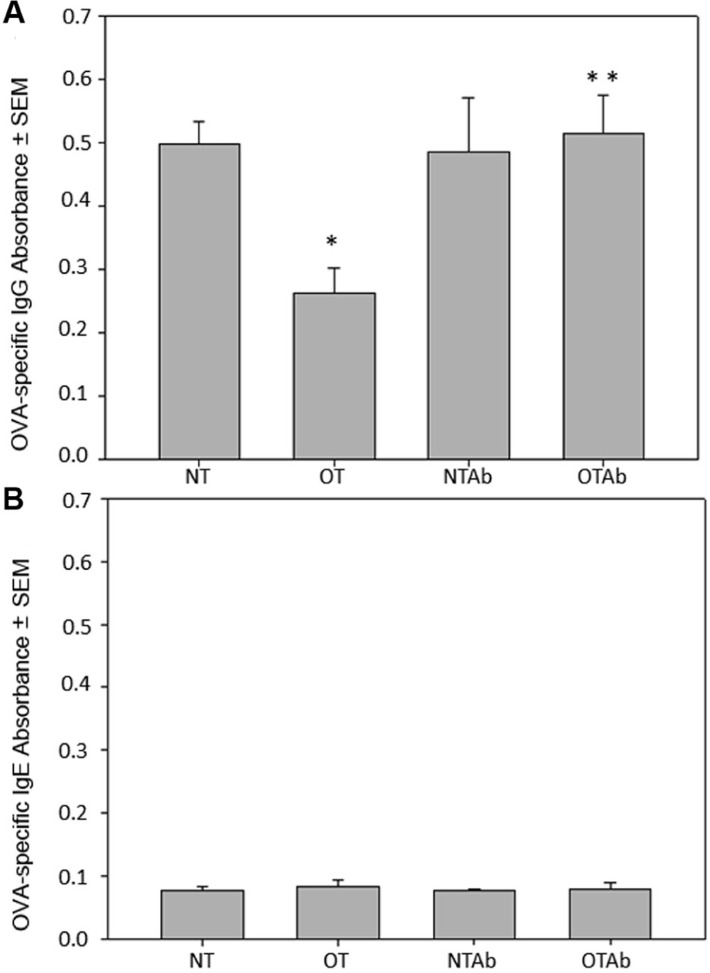
**Levels of OVA-specific IgG are altered in response to oral administration of OVA following erythromycin treatment.** Mice were divided into four treatment groups (*n* = 3–4/group). Mice were administered water (NT and OT) or 5 mg of the antibiotic erythromycin (NTAb and OTAb) for 6 days, followed by 20 mg of OVA (OT, OTAb) or water (NT, NTAb) on day 7. At 5 and 12 days post-OVA treatment, all mice were immunized with 0.1 mg of OVA. Seven days after the second immunization, blood serum was isolated and serum levels of OVA-specific IgG **(A)** and OVA-specific IgE **(B)** were determined by ELISA (NT compared to OT *p ≤ 0.05, OT compared to OTAb **p ≤ 0.05 as determined by one-way ANOVA followed by Student–Newman–Keuls *post hoc* analysis). Data presented are of a representative experiment of three total experiments.

Antibiotic treatment has been implicated in the development of various autoimmune diseases in humans such as allergy, asthma (Wickens et al., 1999; Droste et al., 2000; Marra et al., 2009; Russell et al., 2012), and Crohn’s disease (Card et al., 2004). Given the importance of generating tolerant immune responses to prevent the initiation of autoimmune responses and subsequent disease, we investigated the effect of antibiotic treatment on oral tolerance induction. Following antibiotic treatment (NTAb and OTAb), administration of a single dose of OVA (OT and OTAb), and immunization with OVA (all mice), blood serum was isolated to determine levels of OVA-specific IgG. Levels of OVA-specific IgG were reduced in the tolerized (OT) group compared to the non-tolerized (NT) group, demonstrating that the tolerizing procedure was effective (**Figure 1A**). However, in the OTAb group OVA-specific IgG was markedly elevated from the tolerized control, and very similar to the IgG levels observed in the NT control group (**Figure 1A**). OTAb levels of OVA-IgG that are comparable to the NT group demonstrate an inability to suppress an antibody response to OVA, suggesting that tolerance to OVA was not achieved in the antibiotic-treated group.

It has previously been demonstrated that germ-free mice produce IgE, generating allergic reactions to orally fed antigens, but replacement of individual intestinal microbial species restores oral tolerance (Tanaka and Ishikawa, 2004). Furthermore, a similar investigation into the effects of antibiotic treatment on tolerance examined levels of IgE and found no effect of antibiotic treatment on whey protein-specific IgE. Blood serum from the four treatment groups was isolated as described above. Levels of OVA-specific IgE were not detected above background levels in any of the treatment groups (**Figure 1B**), demonstrating that an IgE response was not generated to OVA in our model system.

### ANTIBIOTIC TREATMENT REDUCES THE INTESTINAL *LACTOBACILLUS* POPULATION

**FIGURE 2:**
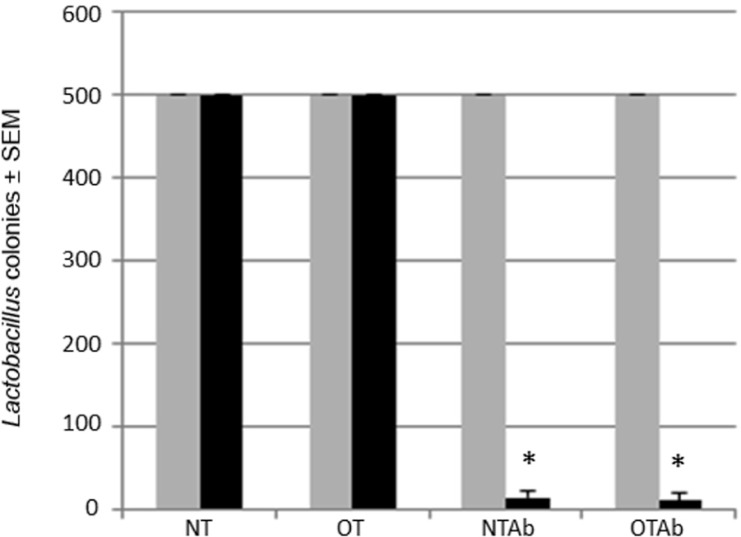
**Erythromycin reduces the *Lactobacillus* probiotic population in the intestines of mice.** Mice were administered 5 mg erythromycin (NTAb and OTAb) or water (NT and OT) once a day for seven consecutive days via intragastric gavage. Fecal samples were collected from each mouse pre- (gray bar) and post-treatment (black bar). Samples were plated on MRS agar plates, and Lactobacilli colonies were enumerated following a 24-h incubation at 37^°^C and 10% CO_2_ (500 colonies per plate was the maximum number of colonies counted). *p ≤ 0.05 by paired *t*-test. Data presented are of a representative experiment of three total experiments.

One major complication of antibiotic treatment is its alteration of intestinal microbial communities (Hoban, 2003). Altered intestinal microflora have been found to have a role in irritable bowel disorders (Frank et al., 2007) and atopic diseases (Wickens et al., 1999; Droste et al., 2000; Bjorksten et al., 2001). *Lactobacillus* is a model probiotic genera (Ivanov and Littman, 2011) and can be specifically selected for with MRS agar plates. To identify alterations to the probiotic population following antibiotic treatment, the intestinal *Lactobacillus *population was enumerated before and after a 7-day treatment with the broad-spectrum antibiotic erythromycin to determine the effect of the antibiotic treatment on the intestinal probiotic population of mice. Erythromycin was chosen because of its ability to reduce enteric bacteria populations, including *Lactobacillus* (Finegold, 1970; Andremont et al., 1983). Fecal samples collected pre- and post-treatment demonstrated that erythromycin reduced the populations of Lactobacilli in the GI tract (**Figure 2**).

### TOLERANCE INDUCTION FOLLOWING ANTIBIOTIC TREATMENT ALTERS POPULATIONS OF CD11c^+^/CD11b^+^/CD8α^-^ TOLEROGENIC DCs IN THE MLNs, BUT NOT OTHER TOLEROGENIC DC SUBSETS

**FIGURE 3:**
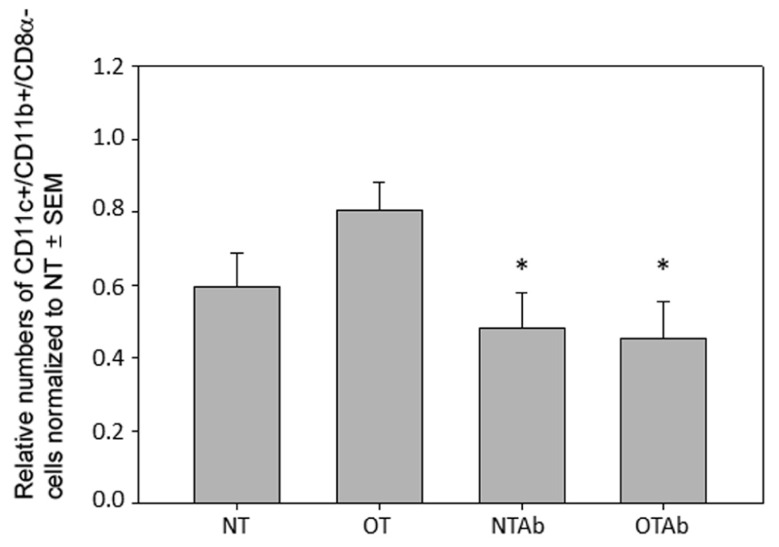
**Antibiotic treatment alters populations of CD11c^+^/CD11b^+^/CD8α^-^ DC populations in the MLN**. Erythromycin (5 mg) was administered daily to NTAb and OTAb mice and water given to NT and OT mice. On day 7, 20 mg of OVA was administered in water (OT) or antibiotic solution (OTAb) to induce tolerance. MLNs were harvested on day 33 (following treatment plan described in Section “Materials and Methods”) and cell suspensions were stained with antibodies for the described surface proteins. Populations were enumerated using an Accuri C6 flow cytometer. Data were normalized to the NT group, pooled from three experiments, and analyzed by one-way ANOVA followed by Student–Newman–Keuls *post hoc* analysis for significance (*p ≤ 0.05 compared to OT).

To explore the mechanisms behind this hindrance of tolerance induction, subsets of tolerogenic DCs were examined. While tolerogenic DC subsets differ between various blood and lymphoid tissues, the tolerogenic subsets found in the MLNs are CD11c^+^/CD11b^+^/CD8α^-^ DCs, CD11c^+^/CD103^+^ DCs, and CD11c^+^/MHC Class II^+^/CD103^+^ DCs (Niess and Reinecker, 2006; Tezuka and Ohteki, 2010; Scott et al., 2011). Examination of CD11c^+^/CD11b^+^/CD8α^-^ DCs 2 weeks after tolerance was induced revealed no significant increase of this population in the MLNs of tolerized mice compared to non-tolerized mice, however, both tolerized and non-tolerized antibiotic-treated groups had a significant decrease in the CD11c^+^/CD11b^+^/CD8α^-^ population as compared to the OT group (**Figure 3**), demonstrating that antibiotic treatment alone may be altering populations of tolerizing DCs in the MLNs.

**FIGURE 4:**
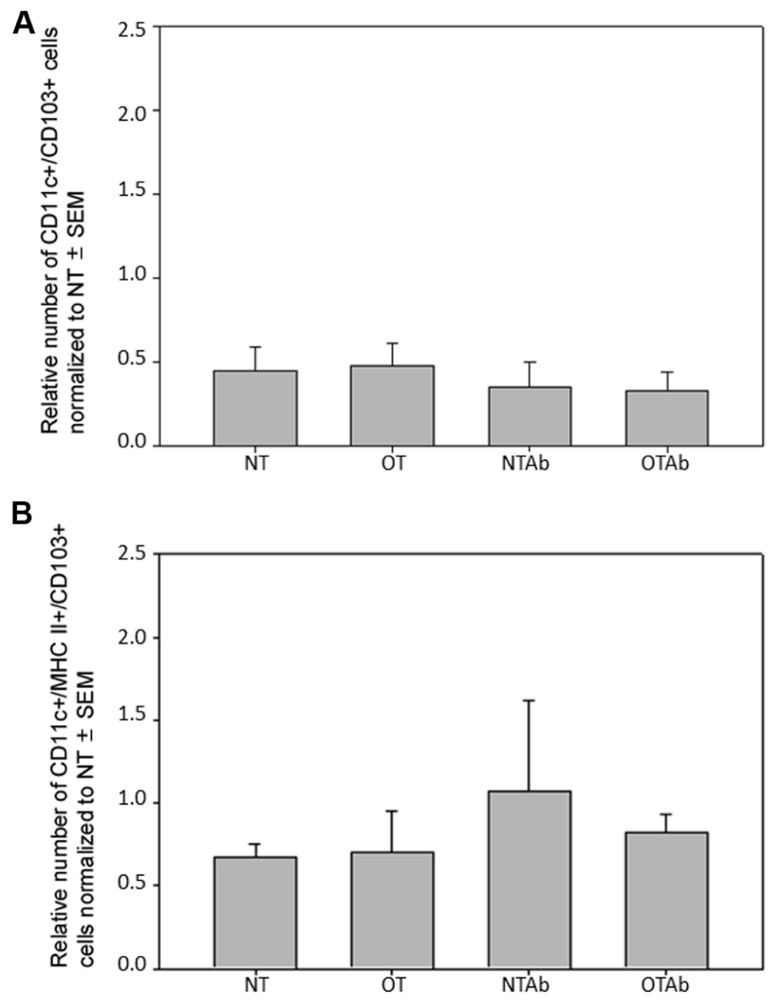
**Antibiotic treatment does not alter populations of CD11c^+^/CD103^+^ or CD11c^+^/MHC Class II^+^/CD103^+^ DC populations in the MLNs**. Erythromycin (5 mg) was administered daily to NTAb and OTAb mice and water given to NT and OT mice. On day 7, 20 mg of OVA was administered in water (OT) or antibiotic solution (OTAb) to induce tolerance. MLNs were harvested on day 33 (following treatment plan described in Section “Materials and Methods”) and cell suspensions were stained with antibodies for the described surface proteins. CD11c^+^/CD103^+^ **(A)** or CD11c^+^/MHC Class II^+^/CD103^+^ **(B)** DC populations were enumerated using an Accuri C6 flow cytometer. Data were normalized to the NT group, pooled from two experiments, and analyzed by Kruskal–Wallis ANOVA on ranks for significance.

To further investigate the effect of tolerance and antibiotic treatment on other DC populations in the MLNs, populations of CD11c^+^/CD103^+^ DCs and CD11c^+^/MHC Class II^+^/CD103^+^ DCs were examined. There were no significant differences between treatment groups for CD11c^+^/CD103^+^ DCs (**Figure 4A**) or CD11c^+^/MHC Class II^+^/CD103^+^ DCs (**Figure 4B**). To identify any alterations that may occur shortly after tolerance induction, all three MLN DC subset populations were enumerated 24 h after oral administration of OVA. No significant differences between treatment groups was observed in any of the DC populations at this time point (data not shown). Taken together, these data suggest that there is no direct association between the inability to induce tolerance after reduction in intestinal Lactobacilli and tolerogenic DC populations in the MLNs, although antibiotic treatment alone may be sufficient to decrease some tolerogenic DC populations.

### TOLERANCE INDUCTION FOLLOWING ANTIBIOTIC TREATMENT DOES NOT ALTER POPULATIONS OF Tregs IN THE MLNs

**FIGURE 5:**
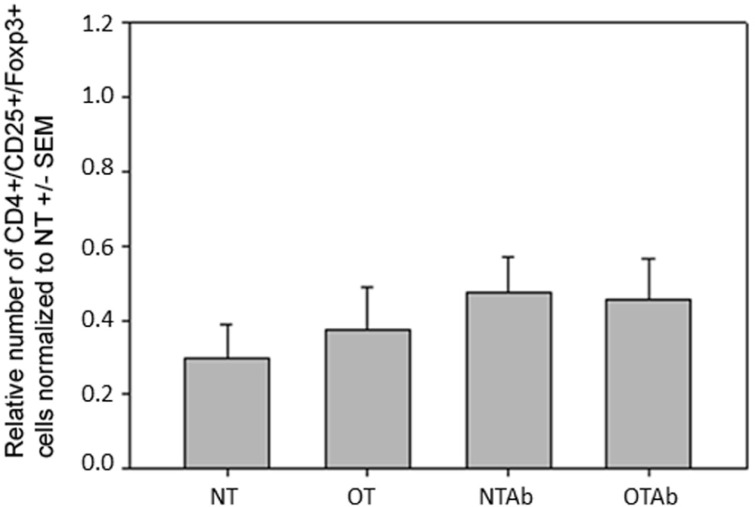
**Antibiotic treatment prior to oral administration of OVA does not alter CD3^+^/CD4^+^/CD25^+^/FoxP3^+^ Treg populations in MLN**. Mice were administered water (NT and OT) or 5 mg of the antibiotic erythromycin (NTAb and OTAb) for 6 days, followed by 20 mg of OVA (OT, OTAb) or water (NT, NTAb) on day 7. MLNs were harvested on day 33 (following treatment plan described in Section “Materials and Methods”), cell suspensions were stained with antibodies for the described surface proteins, and intracellular staining was performed for the transcription factor FoxP3. Populations were enumerated using an Accuri C6 flow cytometer. Data were normalized to the NT group, pooled from three experiments, and analyzed by one-way ANOVA for significance.

Because DCs in the MLNs have a primary role in naïve T cell differentiation, specifically Treg differentiation, CD3^+^/CD4^+^/CD25^+^/FoxP3^+^ Treg populations in the MLNs were enumerated. There were no differences observed in the MLN Treg populations between the different treatment groups 14 days (**Figure 5**) or 24 h (data not shown) after tolerance induction, suggesting there is no direct link between alterations in total Treg populations and the hindrance of tolerance following antibiotic treatment.

## DISCUSSION

Given the emerging evidence highlighting the importance of probiotics and the normal intestinal flora in health and disease, this study sought to examine alterations in immune mechanisms brought about by antibiotic treatment. The data presented in this study demonstrate that treatment with erythromycin prior to the oral introduction of antigen hinders the induction of oral tolerance, such that mice tolerized to antigen after antibiotic treatment have an immune response that is comparable to non-tolerized controls, and this may be associated with the alteration in normal intestinal flora due to antibiotic treatment, as intestinal populations of Lactobacilli were reduced following erythromycin treatment. Furthermore, this study examined populations of DCs and Tregs in the MLNs to identify changes in those cellular populations that may correlate with the inability to induce tolerance in the antibiotic-treated mice. Although no significant alterations in DC or Treg populations were identified following antibiotic and tolerance treatments, there was a decrease in tolerogenic CD11c^+^/CD11b^+^/CD8α^-^ MLN DCs in both the tolerized and non-tolerized antibiotic-treated mice, indicating that antibiotic treatment alone may be sufficient to alter intestinal mucosal DC populations. These are novel studies that provide evidence of antibiotic effects that exceed “common” side-effects and can contribute to the breakdown of normal tolerant immune responses.

The effects of antibiotic treatment on oral tolerance have been examined previously. Pecquet et al. (2004) examined the effect of antibiotic treatment on tolerance induction by assessing levels of β-lactoglobulin (BLG)-specific serum IgE in mice tolerized to whey protein during antibiotic treatment with norfloxacin or gentamicin plus vancomycin. They demonstrated that at the early stages of oral tolerance induction, depleting the gut microbiota with antibiotics weakened the maintenance of oral tolerance to BLG. While tolerance was achieved in antibiotic-treated mice, the more the gut microbiota were altered by antibiotic treatment, the less the tolerant state persisted over time (30–60 days). Findings from our study compliment that of Pecquet et al. by examining other aspects of the humoral response, such as the IgG response. Importantly, our findings demonstrate the ability of erythromycin to abrogate tolerance induction, which is in contrast to the prior findings that tolerance could be induced and antibiotic treatment only prevented maintenance of a tolerant state. Furthermore, the results from our study revealed no differences in IgE levels between treatment groups, which is likely a result of the different model systems such that OVA may not elicit the atopic, IgE-producing response that whey protein does. Importantly, both our study and that of Pecquet et al. demonstrate a reduction in intestinal microflora that correlates with alterations in either the induction or maintenance of a tolerant state.

The role of probiotics in oral tolerance induction has been debated, and this study provides further information for this debate. Furrie et al. (1995) fed germ-free mice with OVA and analyzed OVA-specific IgG levels and systemic delayed-type hypersensitivity (DTH) responses to conclude that the absence of intestinal microbiota has no effect on oral tolerance induction to OVA. In contrast, a study by Prioult et al. (2003) demonstrated that in germ-free mice, oral tolerance to BLG cannot be achieved; however, reconstitution of gut flora with only one microbial species is sufficient to permit oral tolerance induction and subsequent suppression of humoral and cellular responses, providing evidence that probiotics modulate oral tolerance responses in mice. Our studies support the prior findings on the importance of probiotics in oral tolerance induction.

This study sought to identify alterations in an immunologic mechanism(s) that correlates with a hindrance in oral tolerance induction. No changes in the MLN Treg populations were observed between treatment groups (**Figure 5**), indicating that there is no correlation between alterations in the Treg populace and the hindrance of tolerance in the antibiotic-treated group. However, there are two limitations to our study that may contribute to this finding. First, total Treg populations, and not OVA-specific populations, were examined. It is possible that alterations in the OVA-specific Treg population exist while total Treg populations remain unchanged. Additionally, it is possible that the method of oral tolerance induction in our study design contributed to a lack of observable change in Treg populations. Our study utilized the high-dose method for tolerance induction. While this was the best method of inducing tolerance in our model, it can be argued that this mechanism for initiating oral tolerance leads to the deletion of autoreactive cells and of Tregs. It has previously been demonstrated that the feeding dosage of antigen has a profound effect on the mechanism by which oral tolerance is achieved. While animals fed with a low-dose (1 mg) of antigen over several days induced tolerance via active suppression of immune cells and increased secretion of the TGF-β, animals fed with a one-time high-dose (20 mg) of antigen induced tolerance by anergy and an increased secretion of the cytokine IL-4 (Friedman and Weiner, 1994). As a result, high-dose tolerance induction affects antibody responses, while low-dose induction affects regulatory cell-mediated responses (Mowat et al., 1983). In our model system, if the high-dose mechanism of tolerance has more effect on antibody responses and promotes anergy instead of initiating the induction of Tregs, this may explain the absence of differences in Treg populations in our treatment groups even though antibody responses to OVA were altered.

Interestingly, antibiotic treatment in both the non-tolerized and oral-tolerized treatment groups resulted in a decrease in CD11c^+^/CD11b^+^/CD8α^-^ MLN DCs (**Figure 3**), suggesting that the antibiotic treatment itself, excluding the tolerizing antigen, has an effect on this tolerogenic DC subset. It is possible that this is due to alterations in the intestinal epithelium as a result of the antibiotic treatment. Disruption of the intestinal probiotic population can result in intestinal alterations and inflammation (Levy, 2000). It is possible that in inflamed intestinal tissue (due to antibiotic treatment), antigen-loaded tolerogenic DCs that would normally be transported to the MLNs may instead enter the blood stream and traffic to tissues other than the MLNs, inducing an active, rather than tolerant, immune response to antigen such as we observe in our antibiotic treated groups (**Figure 1**).

In conclusion, this was a novel study that demonstrated a break in tolerance resulting from a 7-day course of erythromycin that was associated with a decrease in a subset of tolerogenic DCs potentially and may involve alterations in the normal intestinal microflora. Our findings, coupled with the finding that antibiotic treatment-induced alterations of intestinal microflora extend past antibiotic treatment and permanently alter the composition of the microflora (Littman and Pamer, 2011), support previous epidemiologic studies that suggest antibiotic usage may be linked to more serious health conditions including atopic disorders such as allergy and asthma (Wickens et al., 1999; Droste et al., 2000; Russell et al., 2012) and irritable bowel disorders (Card et al., 2004) and provide a basis for further investigation into the effects of antibiotic treatment on immune responses.

## Conflict of Interest Statement

The authors declare that the research was conducted in the absence of any commercial or financial relationships that could be construed as a potential conflict of interest.

## Abbreviations

BLG: β-lactoglobulin
DC: dendritic cell
DTH: delayed-type hypersensitivity
ELISA: enzyme-linked immunosorbent assay
GI: gastrointestinal
MLN: mesenteric lymph node
MRS: de Man–Rogosa–Sharpe
OVA: ovalbumin
PBS: phosphate buffered saline
SEM: standard error of the mean
TGF-β: transforming growth factor β
TNBS: trinitrobenzenesulfonic acid
Treg: T regulatory cell.
